# Influence of Particle Size Distribution on the Performance of Ionic Liquid-based Electrochemical Double Layer Capacitors

**DOI:** 10.1038/srep22062

**Published:** 2016-02-25

**Authors:** Anthony J. R. Rennie, Vitor L. Martins, Rachel M. Smith, Peter J. Hall

**Affiliations:** 1 Chemical and Biological Engineering, University of Sheffield, Sir Robert Hadfield Building, Mappin Street, Sheffield S1 3JD, England, UK; 2 Instituto de Química, Universidade de São Paulo - C.P. 26077, CEP 05513-970, São Paulo, SP, Brazil

## Abstract

Electrochemical double layer capacitors (EDLCs) employing ionic liquid electrolytes are the subject of much research as they promise increased operating potentials, and hence energy densities, when compared with currently available devices. Herein we report on the influence of the particle size distribution of activated carbon material on the performance of ionic liquid based EDLCs. Mesoporous activated carbon was ball-milled for increasing durations and the resultant powders characterized physically (using laser diffraction, nitrogen sorption and SEM) and investigated electrochemically in the form of composite EDLC electrodes. A bi-modal particle size distribution was found for all materials demonstrating an increasing fraction of smaller particles with increased milling duration. In general, cell capacitance decreased with increased milling duration over a wide range of rates using CV and galvanostatic cycling. Reduced coulombic efficiency is observed at low rates (<25 mVs^−1^) and the efficiency decreases as the volume fraction of the smaller particles increases. Efficiency loss was attributed to side reactions, particularly electrolyte decomposition, arising from interactions with the smaller particles. The effect of reduced efficiency is confirmed by cycling for over 15,000 cycles, which has the important implication that diminished performance and reduced cycle life is caused by the presence of submicron-sized particles.

With a growing need for electrical energy storage devices there has been a substantial volume of recent research focused on the understanding and development of electrochemical capacitors (ECs)^1^,^2^,^3^,^4^,^5^,^6^. Also referred to as supercapacitors, these devices are able to accept and deliver charge rapidly through the exploitation of fast redox reactions at an electrode surface (pseudocapacitors) or through the storage of charge at the interface between an electrode and electrolyte (electrochemical double layer capacitors, EDLCs)^7^. Currently, EDLCs represent 80% of commercially available ECs and are known for their reliability^2^, high power density (>10 kW kg^−1^) and long cycle lives (>100,0000)^7^.

The large capacitance of EDLCs arises from the reversible adsorption of electrolyte ions at the interface with high surface area electrode and as a result carbon materials are usually employed as the main electrode component as they couple relatively high specific surface areas with good electrical conductivity and electrochemical stability. There are a substantial number of reviews available concerning the influence that chemical and structural properties of the carbon material have on the electrochemical performance of EDLC electrodes [*e.g.* refs. 1,2,8, 9, 10].

EDLC electrodes typically consist of a composite layer of activated carbon particles with an organic polymer (such as PTFE or PVdF) or carboxymethyl cellulose bound to a metallic current collector^11^. Each of these components contributes to the equivalent series resistance (ESR) of devices as well as the resistance offered by the electrolyte and the interface between the electrode and electrolyte. Resistance can be considered to be a combination of the resistances associated with electron transport in the electrode as well as ion transport in the electrolyte and limits the maximum output power and rate performance of EDLCs.

When considering only the resistivity of powdered carbon in a packed bed, the electrical resistance is a function of the intraparticle, (*i.e.* intrinsic) and interparticle (or ‘contact’) resistances. Intraparticle resistance is dependent on the degree of graphitization and microstructure present in the particle. Interparticle resistances are dependent on the morphology of the particles and the compaction pressure exerted on the bed. The size, shape and distribution of particle sizes influences the packing in the bed and therefore the number of interactions between particles, and this in turn can affect the bed resistivity. It has been reported that when using different particle size classifications of the same carbon material, an increase in resistivity with decreasing particle size is encountered which arises from a greater number of contacts per unit length^12^.

EDLC electrodes comprising a composite coating can be considered as conductive particles contained within an insulating matrix and the contributions to the ESR arise from the binder material and interparticle contact resistances. In addition these coatings include void space not only from the intrinsic porosity of the carbon particles but arising from the interstitial space between particles. Such voids are necessary to facilitate electrolyte access but also contribute significantly to device resistance.

Particle packing is difficult to predict, especially for powders containing complex mixtures of non-spherical particles such as the materials used in this study. In general, reducing particle size results in increased voidage^13^,^14^. This effect is most apparent for particles under 100 μm in size, due to the effect of long-range interparticle forces, which hinders the relative movement of fine particles. It has been shown that an increase in the fraction of fine particles leads to higher values of voidage^13^. The breadth of the particle size distribution can also have an effect on the voidage, with research showing that an increase in the particle size distribution breadth reduces total voidage^15^, but produces broader void size distributions^16^,^17^. Due to the effect of particle size on total void volume and void sizes, the particle size distribution can be expected to influence the resistance and performance of devices using composite electrodes. Figure 1 illustrates the differences in packing that could be expected for different particle size distributions.

In the few reports concerning the influence of particle size on the performance of EDLC electrodes it is generally found that reducing the average particle diameter results in improved capacitance and rate performance. Yoshida *et al.*^18^ found that the internal resistance of EDLCs increased and that capacitance decreased with increasing average particle size (3.5–18.1 μm). This behavior was attributed to denser packing of activated carbon occurring at smaller particle sizes and it was noted that volume of voids increased substantially with particle size. The same trends in resistance and specific capacitance were reported by Portet *et al.*^19^ over a wider range of average particle sizes (0.02–20 μm) that included nanoparticles. It was shown that, in general, EDLCs based on larger particle sizes exhibited poorer capacitance retention with increasing discharge rate. The reduction in particle size facilitates ion transport by reducing the diffusion length of the ions. Similar behavior has also been reported^20^ for monodisperse carbon spheres with average diameters in the sub-micron range.

These reports focus on ideal particle size distributions represented by a singular characteristic such as average particle size. However, the particle size distribution can contain a broad range of particles sizes, and it is difficult to compare different populations of particles using a single particle size descriptor. Also, these previous investigations study electrochemical behavior of electrodes using an aqueous or organic solvent-based electrolyte. In order to improve the energy density of EDLCs a substantial amount of research has investigated the use of ionic liquid electrolytes which permit the use of wider operating potentials^11^,^21^,^22^,^23^ but are known to adversely influence power density and to behave in a different manner to conventional electrolyte systems.

In this report we investigate the influence of particle size distribution on the performance of EDLC electrodes using an ionic liquid electrolyte. Composite electrodes are coupled with *N*-butyl-*N*-methyl pyrrolidinium bis(trifluoromethanesulfonyl) imide (a widely investigated ionic liquid capable of stable operation at potentials in the region of 3.5–3.7 V^24^,^25^) and their performance in terms of specific capacitance, resistance and coulombic efficiency considered with respect to the particle size distributions of the activated carbon material.

## Results and Discussion

### Physical characterization

Particle size distributions of the milled samples were determined using laser diffraction and are expressed as volume density plots in Fig. 2(a). The materials tend to show a bimodal particle size distribution, with the presence of a peak in the 1–5 μm region and another at 10–50 μm. The larger peak also shifts to lower sizes with increasing milling time. In general, as the milling duration increases the volume of large particles decreases with a corresponding increase in the fraction of particles less than 5 μm in size. Two exceptions to this trend are observed with the samples milled for 30 and 90 min; less pronounced peaks than expected in the 1–5 μm region were observed. This could be attributed to the possible ingress of oxygen during milling or the disproportionate loss of fines when handling the samples.

In the case of the sample milled for 90 min this apparent loss of smaller particles could be attributed to agglomeration of smaller particles which is indicated by the small peak seen in the particle size distribution at a size of 40–70 μm. (This peak is also evident to lesser extents in the samples milled for 45 and 60 min.) Agglomeration may also be responsible for the unexpected reduction in the 1–5 μm peak observed for C-30 as the 10–50 μm peak displays a slightly larger volume density than anticipated. It is possible that smaller particles (*ca.*1 μm) form agglomerates that are of similar size to the primary particles.

**Figure 1:**
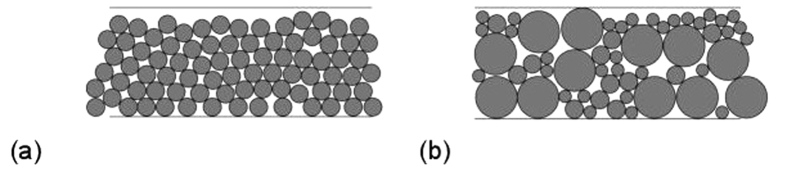
Expected packing for (**a**) mono-sized spheres and (**b**) a broad particle size distribution.

To illustrate the breadth of the particle size distribution several descriptors of particle size derived from the data in Fig. 1(a) are presented in Table 1. As could be expected, the various measures of particle size in Table 1 are seen to decrease with increasing milling duration and the fraction of particles below 1 μm is seen to increase. The effects of particle size, volume and surface area on electrode performance are complex, and due to this it is difficult to choose a single particle size descriptor to use for comparison between different particle size distributions. The surface-volume mean, D_[3,2]_, may be most appropriate as it retains the surface area and volume of the original distribution^26^. The surface-volume ratio of the particles may be related to the number of interparticle contacts per mass, and therefore to the resistivity of the electrode.

**Figure 2:**
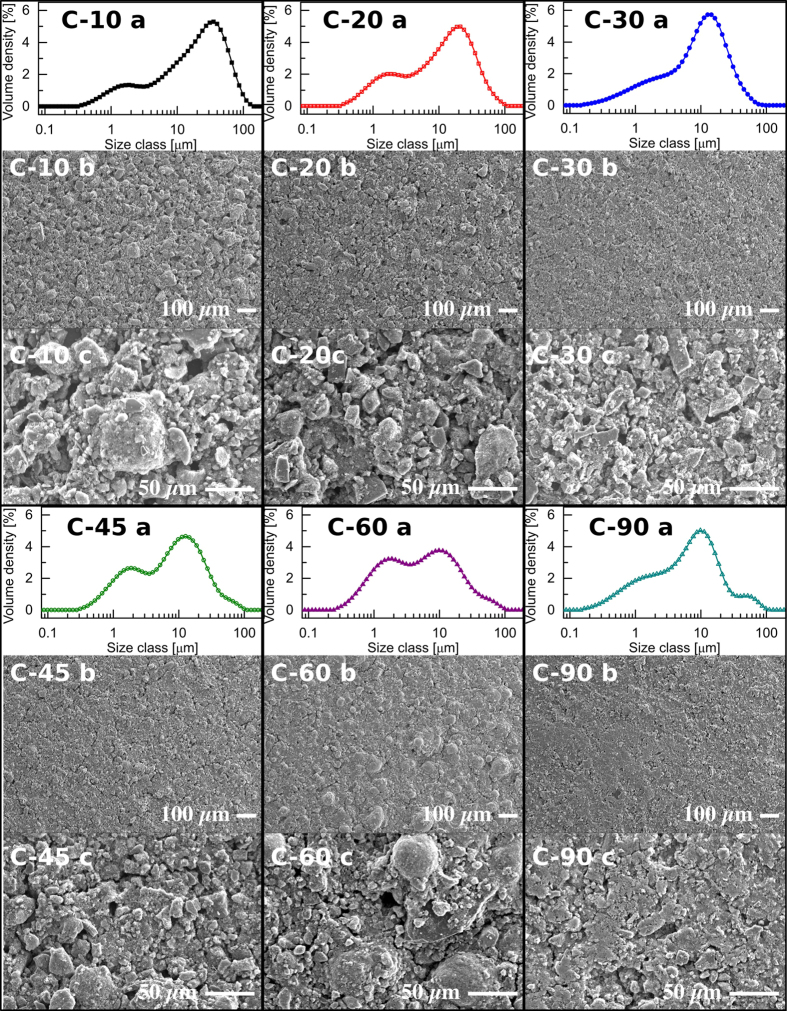
(**a**) Particle size distributions of the milled powders (**b**) SEM images of electrode coatings at ×200 magnification and (**c**) at ×500 magnification.

The change in particle size distribution is evident in the SEM images of the electrode surfaces shown in Fig. 2(b,c) (lower and higher magnifications). In Fig. 2(b) it is clear that the fraction of smaller particles increases in relation to the larger particles as the milling duration increases. As the milling duration increases from 10 min to 45 min the appearance of the electrode surfaces Fig. 2(b) becomes smoother which can be attributed to both the reduction in average particle size and the increasing fraction of smaller particles present.

An exception to this is seen for C-60 electrodes, which appear rougher due to the presence of several spherical particles distributed around the surface. (Detailed SEM images of the surface are provided in Fig. 1S in the Supplementary Information (SI).) It is likely that these spheres are an artifact of the electrode manufacture process rather than primary particles as they are only evident on the C-60 electrodes. Considering the electrode manufacture process where carbon and polymer binder are dispersed in acetone and stirred, it is likely that spherical agglomerates form and are incorporated into the electrode coating. (It is noted that the surface of C-90 electrode appears slightly compacted and that such spheres may have been present but were compressed during handling.)

The homogenous distribution of binder in the electrodes was confirmed by EDX mapping of fluorine (present in the Kynar co-polymer). EDX mapping of fluorine indicates that the binder forms a thin layer on the surface of all of the carbon particles rather than existing in the form of tendrils or large polymer rich regions. Micrographs for all electrodes along with EDX mapping of carbon and fluorine are provided in Fig. 2S of the SI.

Table 1 also includes surface area and pore characteristics determined from nitrogen adsorption/desorption isotherms (see Fig. 3S in SI) obtained for the milled carbon materials at −196 °C. The specific surface area of the materials as determined by the BET method is seen to decrease with milling time with the exception of C-30, a trend which is also seen in total pore volume and micropore volume. As particles are likely to fracture along the largest existing pores during milling, a steady reduction in pore volume and surface area may be anticipated. However, it is possible that in addition to the porosity measured by nitrogen, the particles contain some inaccessible (or “closed”) porosity that is opened during the milling process. The competing effects of pore destruction and opening may be responsible for the reduced surface area, micropore volume and total pore volume of C-30 when compared with samples milled for a longer period.

Strangely, the trend observed in mesopore volume (pores with a width in the region of 2–50 nm) does not seem to follow the same trend as seen in total pore volume or micropore volume. Mesopore volume is seen to decrease from a value of 0.27 cm^3^g^−1^ for C-10 and C-20 to 0.25 cm^3^g^−1^ for C-30 and increases to *ca* 0.30 cm^3^g^−1^ in the case of C-45 and C-60. This again indicates that two competing processes with respect to pore volume reduction/creation are occurring during milling.

### Electrochemical characterization

**Figure 3:**
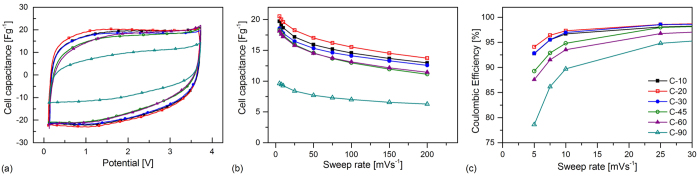
(**a**) Cyclic voltammograms of the cells at 25 mVs^−1^, (**b**) cell capacitance with sweep rate determined using cyclic voltammetry, and (**c**) coulombic efficiency of cyclic voltammograms up to 25 mVs^−1^ for the milled carbon materials.

Figure 3(a) shows cyclic voltammograms obtained at 25 mVs^−1^ for cells using each of carbons studied. It is clear that they are all of similar rectangular form and the absence of any significant changes in response with changing potential indicates that the double layer mechanism is responsible for charge storage. Also it is evident that there is little difference in the magnitude of the response for all cells besides those employing C-90 under these experimental conditions. This could be anticipated as C-90 exhibits a substantially lower specific surface area than the other materials (Table 1).

The slight deviation from ideal rectangular behavior seen with cells incorporating C-45 and C-60 indicates increased resistances associated with these electrodes, which can be expected to adversely impact their rate performance. This is seen clearly in Fig. 3(b), which summarizes the behavior of the cells over a range of sweep rates. Each of the samples displays behavior typical of EDLCs, where a maximum cell capacitance is attained at the lowest rate and decays with increasing rate.

At the lowest rate studied (5 mVs^−1^) C-20 generates the largest cell capacitance of 20.5 Fg^−1^ and produces the highest cell capacitances of all samples at each of the sweep rates studied. This is perhaps an unexpected result if only the specific surface areas of the samples are considered as C-10 displays a slightly larger surface area and micropore volume than C-20 (Table 1). In addition, if only the surface area and porosity characteristics were considered it would be expected that C-30 would be associated with lower specific capacitances than C-45 and C-60. As this was not observed this indicates that the differences in particle size distribution rather than porosity are responsible for the differences in behavior observed.

However, it should be noted that electrodes produced using C-10 were observed to be fairly fragile in comparison with the others despite the relatively high proportion of binder used in their construction. This is evident in Fig. 2(b) where the SEM images show that the C-10 electrode has a much rougher surface in comparison with the others, as well as possessing a significant number of large pores. Although this is a fairly subjective analysis it is unlikely that such electrodes would be useful in commercial devices as their poor mechanical stability renders them unsuitable for winding, and the cutting process often resulted in significant portions of the coating becoming detached from the current collector. Consequently, it is possible that some of the active material in these electrodes is isolated from the current collector and unable to participate in the charge storage processes. This reasoning is supported by the fact that a substantially higher deviation between cells utilizing C-10 was found in comparison with that associated with cells based on other materials. (At a sweep rate of 25 mVs^−1^, the cell capacitance of C-10 was within a tolerance of ±8.9% whereas all other materials were within ±3.0%.) As the larger modal particle size of C-10 is substantially greater than that of the other samples this is likely to be a crucial parameter in electrode manufacture and suggests the existence of a critical particle size for the production of reliable, robust coatings when using the same proportion of binder and coating setup. It is likely that this critical size is related to the thickness of the electrode.

Excluding C-10, the specific capacitance at a sweep rate of 5 mVs^−1^ decreases with increasing milling duration and D_[3,2]_; this trend is observed until a sweep rate of 50 mVs^−1^. Although there is little difference between the performance of the C-45 and C-60 electrodes, slightly better performance at rates in excess of 50 mVs^−1^ is found with C-60 which in indicative of a lower internal resistance. Considering the SEM images in Fig. 2(b,c) it is possible that spherical agglomerates seen in the C-60 electrodes are responsible for this behavior. If these aggregates are composed mainly of binder and a number of smaller particles, a fraction of the smallest particles are isolated from the bulk electrode in such aggregates. This results in substantially fewer interparticle contact resistances rather than the increase that could be anticipated by considering particle size distribution alone.

Loosely, the general trend in measured specific capacitances under given conditions is an observed decrease with decreasing average particle size. This is in contrast with the previously discussed reports where the opposite trend was found^18^,^19^,^20^. There are several possible reasons for this apparent discrepancy. For instance the particle size distribution in this work is known to be bimodal whereas there is no information available regarding this characteristic for the other works. Also, it is known that in this work the surface area decreases (Table 1) with increasing milling time and in other reports^19^,^20^ surface area is seen to decrease with increasing particle size.

The coulombic efficiency for the cyclic voltammograms summarized in Fig. 3(b) was determined at each of the sweep rates and were found to increase with increasing rate with all samples exhibiting efficiencies greater than 99% at rates greater than 100 mVs^−1^. The coulombic efficiency at sweep rates up to 25 mVs^−1^ are illustrated in Fig. 3(c).

Interestingly, the coulombic efficiency of cells at low sweep rates is seen to reduce with increasing milling duration. Losses in efficiency are typically caused by charge participating in side reactions or self-discharge processes. A possible side reaction encountered in EDLCs is electrolyte decomposition that occurs when the electrode potential is beyond the electrochemical stability limit of the electrolyte. This is suggested by the upturn of the current response during charging potentials approaching 3.6 V in Fig. 3(a) and is particularly evident in cells using C-90. From the characteristics given in Table 1 it can be seen that the volume of the finest particles (less than 1 μm) expressed as a percentage of the total volume increases with milling duration. As reductions in coulombic efficiency correspond to an increasing proportion of smaller particles it is proposed that this fraction is responsible for accelerating the decomposition of electrolyte at higher potentials.

In addition to reducing the average particle size and generating an increasing fraction of fine particles, the attrition of large particles during the milling process can be expected to expose more graphitic edges and produce broken carbon-carbon bonds that are highly reactive^28^,^29^. These sites react on exposure to atmospheric oxygen resulting in surface functionalities that may be able to participate in side reactions with electrolytes. It can be anticipated that the concentration of edge sites and surface functionalities increase with milling duration and that these are responsible for the reduction in coulombic efficiency by participating in side reactions or electrolyte decomposition.

As the results associated with C-10 and C-90 presented above suggest that they are of limited practical use, for the reasons of poor mechanical stability (C-10) and substantially diminished capacitive performance (C-90), they are excluded from further consideration. Table 2 provides specific capacitance values (c_cv_) determined from Fig. 3(a) for the materials under consideration.

**Figure 4:**
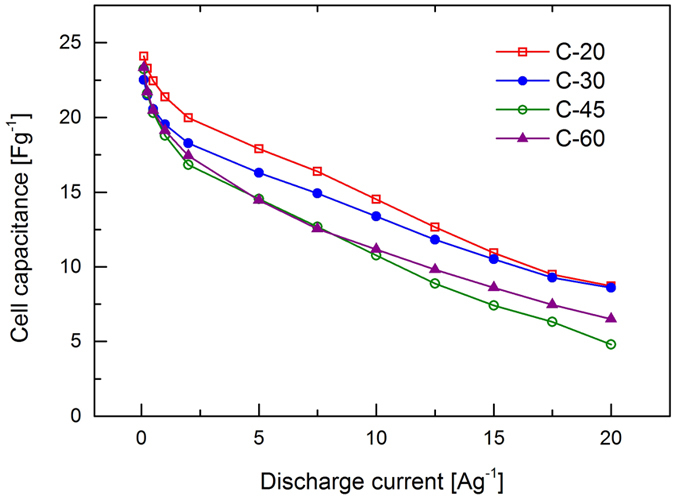
Cell capacitance determined at different rates of constant current discharge between 3.6 V and 0 V.

A wider range of charge/discharge rates was explored using galvanostatic cycling at specific currents between 0.1 and 20 Ag^−1^ and the results are presented in Fig. 4. Cell capacitances at rates of 0.5 Ag^−1^ (c_0.5_) and 5 Ag^−1^ (c_5.0_) from Fig. 4 are given in Table 2.

At rates lower than 10 Ag^−1^ the same behaviour as seen in Fig. 3(b) is observed, whereas at higher rates (more likely to be experienced in real devices) the internal resistance of the cell has a more pronounced effect on performance. The difference in specific capacitance between C-20 and C-30 decreases with increasing rate with near identical performance observed at 20 Ag^−1^. Although C-20 exhibits the highest specific capacitance at low rates it is likely that this arises from the utilisation of the smallest pores which have insufficient time to participate in the charge storage mechanism at higher rates. This is as expected as C-20 possesses the largest micropore volume and surface area (Table 1).

**Figure 5:**
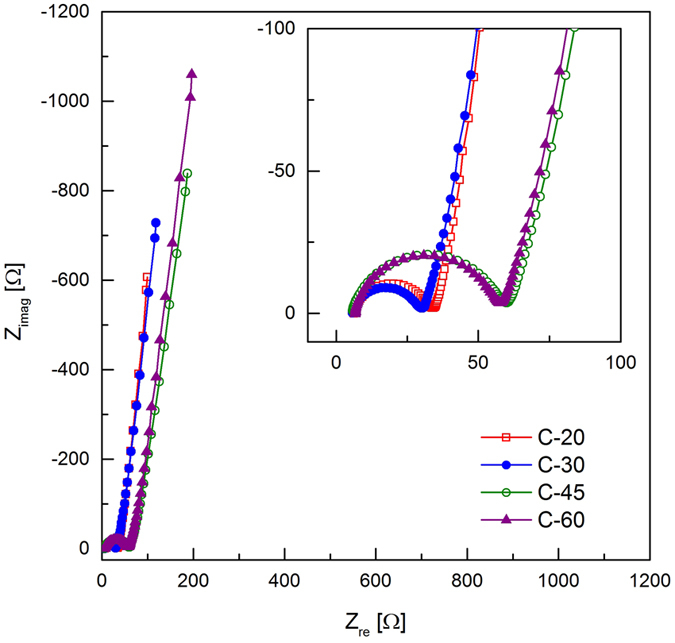
Nyquist plots showing representative data from EIS experiments, magnified high frequency region (inset).

While the specific capacitance of C-20 and C-30 converge with increasing rate, the values associated with C-45 and C-60 are seen to diverge at rates between 10 and 20 Ag^−1^, with C-45 producing significantly lower specific capacitances over this range. This indicates that cells produced with C-20 and C-30 exhibit similar internal resistances and that C-45 and C-60 can be expected to display greater resistances. In order to investigate cell resistance, electrochemical impedance spectroscopy (EIS) was performed on the EDLC cells and example spectra from individual cells are presented in the form of Nyquist plots in Fig. 5.

Each of the spectra describe behavior typical of EDLCs using mesoporous carbon and an ionic liquid electrolyte, displaying a depressed semicircle in the high frequency region (magnified in the inset of Fig. 5) and a linear response in the low frequency range. An ideal capacitive response on a Nyquist plot is represented by a vertical line; the deviation from vertical evident in Fig. 5 for the cells under study indicates a degree of inhomogeneity in the double layer region which is frequently seen when using porous electrodes^30^.

Characteristics derived from the spectra obtained from all cells are given in Table 2. The cell capacitances determined at 10 mHz (C_EIS_) are of the same magnitude as that determined by Galvanostatic discharge at 15–17.5 Ag^−1^.

Values of series resistance (R_s_) are represented by point where the spectra cross the real axis, and are seen to vary little between the cells tested. This is unsurprising considering that this resistance arises due to electrolyte conductivity which remained the same for each cell. In the inset of Fig. 5 it is clear that the behavior of C-20 and C-30 are similar, and that of C-45 and C-60 are nearly identical. The semicircle observed at higher frequencies (R_i_) is attributed to the interactions between the pores of the electrode with the ions in the electrolyte and is sometimes referred to as the cumulative distributed resistance^12^. (R_i_ is related to the ‘transmission line’ equivalent circuit model of resistors and capacitors in parallel used to describe the response of porous electrodes.) For C-45 and C-60 the diameter of the semicircle is far greater than those associated with C-20 and C-30. Considering the characteristics in Table 1 it is seen that the trend in R_i_ loosely correlates with the trend seen in mesopore volume (*i.e.* C-45 > C-60 > C-20 > C-30) and it may be the case that small increases in this measured pore volume substantially influence the observed values of R_i_. It is also possible that interparticle porosity (introduced during electrode manufacture) contributes towards the observed value of R_i_, however the degree to which these interparticle voids influence the measured value of R_i_ remains unclear.

**Figure 6:**
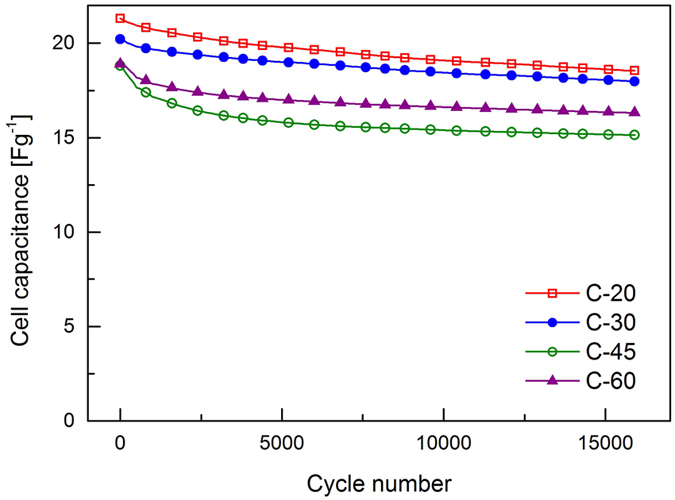
Galvanostatic cycling tests at 1 Ag^−1^ between 0 V and 3.6 V.

Finally, cells were investigated using extended galvanostatic cycling at a rate of 1 Ag^−1^ in order to ascertain whether the particle size distribution has an influence on the lifetime of these devices. Changes in cell capacitance over the first 15,000 cycles are illustrated in Fig. 6. The greatest reduction in cell capacitance is seen in the initial cycles with C-20 and C-30 retaining 97% of the initial capacitance after 1,000 cycles and C-45 and C-60 dropping in performance to a greater extent, exhibiting retentions of 91% and 94% respectively. This correlates roughly with the trend observed in coulombic efficiency (Fig. 3(c)) as the performance of cells with lower efficiency can be expected to fade more rapidly. However, C-60 does not follow the trend and shows better performance than the C-45. As the rate of charge and discharge in used in Fig. 6 is substantially faster than those investigated using cyclic voltammetry it is likely that some regions of all electrodes are electrochemically inactive (accounting for the reduction in cell capacitance observed). As mentioned previously, in the case of C-60 the agglomerates observed using SEM (Fig. 2(b,c)) are likely to be isolated from the electrode and to disproportionately contain the smallest particles. It is proposed that this process results in electrodes comprising C-45 containing the greatest fraction of smaller particles that are electrochemically active and responsible for increased electrolyte decomposition.

## Conclusions

This study shows that the particle size distribution of activated carbons can influence the performance of these materials in composite EDLC electrodes. It appears that increased milling can have an adverse effect on device performance, not only on the specific capacitance and resistance of devices, but also on their efficiency and longevity.

Particle size reduction of activated carbon is required in order to allow materials to be processed in the form of coatings, however it is proposed that a critical particle size exists for the manufacture of homogenous, mechanically stable composite electrodes (likely to be dependent on several factors, one of which being electrode thickness). In this study, milling the activated carbon for 10 minutes was insufficient to produce consistent electrodes. Longer milling durations were found to produce reliable electrodes and their electrochemical performance was found to be influenced significantly by the particle size distribution of the carbon material. In general, increased milling duration resulted in diminished values of cell capacitance over a wide range of charge/discharge rates.

As milling duration increased, the fraction of smaller particles was found to increase which correlates with an observed reduction in coulombic efficiency of EDLCs during cyclic voltammetry at low sweep rates (<25 mVs^−1^) and diminished cycle life of EDLCs. It is likely that the exposure of a greater quantity of graphitic edge groups during milling is responsible for this effect either by accelerating the decomposition of electrolyte or by forming surface functionalities that participate in side reactions during the charging process. This has important implications in the study of new electrolytes, as the physical characteristics of the electrode material have been shown to influence the coulombic efficiency, and will therefore affect the defined operating potential.

In addition, an increasing fraction of smaller particles appears to increase the propensity for agglomeration during processing. In this study the presence of spherical agglomerates produced from the material milled for 60 minutes were found to have the effect of concentrating and isolating this fraction which resulted in better electrochemical performance than could be anticipated, but nevertheless should be avoided in the production of real devices.

This study suggests that a reduction in performance and cycle life is likely to be caused by the presence of submicron sized particles and that it may be beneficial to classify the powders used in the production of EDLC electrodes in order to reduce these effects.

## Methods

Phenolic resin-derived mesoporous carbon beads, 250–500 μm diameter (TE7, MAST Carbon, UK.), were dried under vacuum at 80 °C, before being transferred to a silicon nitride vial (containing two 12.7 mm diameter silicon nitride balls) and sealed under argon in a glovebox. Carbon materials were milled for durations of 10, 20, 30, 45, 60 and 90 minutes in a high energy ball mill (SPEX SamplePrep 8000 M) and were left to cool overnight before exposing to the atmosphere. For ease of reference the carbon materials are referred to by their milling duration *i.e.* C-60 indicates the carbon milled for a period of 60 mins.

Scanning electron microscopy (SEM) images were acquired from composite electrodes using a JEOL JSM-6010LA microscope coupled with an Energy Dispersive X-ray analyser (EDX). Loose particles were removed from the sample surface before analysis. Images were acquired with a working distance of *ca.* 10 mm, and a accelerate voltage of 8 or 10 kV.

Particle size distributions of the milled carbons were measured using a Malvern Mastersizer 3000 laser diffraction particle size analyser. Representative samples of the carbon were dispersed using the Aero S dry powder dispersion system.

Nitrogen adsorption/desorption isotherms at −196 °C were collected using a Micromeritics 3Flex instrument; samples were degassed under vacuum at 200 °C for 10 h before analysis.

### EDLC assembly and electrochemical characterisation

*N*-butyl-*N*-methylpyrrolidinium bis(trifluoromethanesulfonyl) imide ([Pyr_14_] [Tf_2_N]) was purchased from Io-Li-Tec GmbH (Germany) and had a minimum stated purity of >99.5%. Prior to cell assembly [Pyr_14_] [Tf_2_N] was dried through heated stirring for several hours in an argon filled glovebox (H_2_O < 0.1 ppm, O_2_ < 0.1 ppm). Moisture content was determined to be less than 10 ppm using Karl Fischer titration (KF899 Coulometer, Metrohm). EDLC electrodes were produced by spreading a slurry containing carbon material and a polymer binder (KynarFlex^®^ 2801) in an 85/15 ratio (by mass) in the same manner as described in previous reports^31^,^32^. The average carbon loading in a single cell was in the region of 1.6–2.4 mg.

Cell operating potential and electrode mass loading ratio for cells using [Pyr_14_] [Tf_2_N] was determined by considering the efficiency of half-cells during cyclic voltammetry at 5 mVs^−1^ in a manner similar to other reports^33^,^34^. A coulombic efficiency of 97% was used to identify the upper and lower potential limits, which resulted in an operating window of 3.6 V and a mass loading ratio of 1.4 (positive electrode: negative electrode).

Two-electrode button cells (2016) were assembled using stainless steel spacers, carbon based electrodes and glass fibre filter paper separator (GF/F, Whatman) as described elsewhere^31^,^32^.

Cyclic voltammetry was carried out using a Solartron Analytical 1470E Multichannel Potentiostat/Galvanostat between 0 and 3.6 V at sweep rates from 5–200 mVs^−1^. Cells were also cycled galvanostatically between 0 and 3.6 V at various rates between 0.1 and 20 Ag^−1^ (or 1 Ag^−1^ in the case of lifetime determination) using a Maccor 4000 M cell test system. Cell temperature was maintained at 25 °C throughout testing using a Maccor temperature control chamber. Electrochemical impedance spectroscopy (EIS) was performed on the EDLCs at the open circuit potential using a 10 mV perturbation over the frequency range 300 kHz to 10 mHz using a Solartron Modulab XCM.

In the case of cyclic voltammetry experiments the specific capacitance, *C* (Fg^−1^), was determined by considering the quantity of charge delivered during discharge, ∫ *i.dt* (C), the operating potential window, *U* (V), and the mass of active materials in both electrodes, *m* (g) as shown in Equation 1 below.


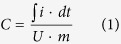


For Galvanostatic measurements, the capacitance was determined from the current, *i* (A) and the slope of the discharge curve (*dV/dt*) after any “iR drop” was observed as shown in Equation 2 below.


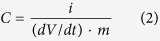


The cell capacitance determined by electrochemical impedance spectroscopy was determined using Equation 3 where *f* represents the perturbation frequency (10 mHz) and *Z*_*imag*_ the imaginary component of the impedance at this frequency.


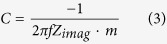


Cell capacitance values are based on the combined mass of carbon material in the electrodes and represent an average of several (at least two) cells of the same composition. Values of specific capacitance determined using cells of identical composition, measured at rates lower than 5 Ag^−1^, are typically within 5.0%. At lower rates, (*e.g.* 5 mVs^−1^) determined values may differ by as little as 2% whereas a larger degree of variation is normally observed rates exceeding 5 Ag^−1^.

## Additional Information

**How to cite this article**: Rennie, A. J. R. *et al.* Influence of Particle Size Distribution on the Performance of Ionic Liquid-based Electrochemical Double Layer Capacitors. *Sci. Rep.* **6**, 22062; doi: 10.1038/srep22062 (2016).

## Supplementary Material

Supplementary Information

## Figures and Tables

**Table 1:** Particle size, surface area and porosity characteristics of milled carbon powders.

Sample	D_V_10^a^ [μm]	D_V_50^a^ [μm]	D_V_90^a^ [μm]	D_[3,2]_^b^ [μm]	D_[4,3]_^c^ [μm]	V < 1 μm^d^ [%]	S_BET_^e^ [m^2^g^−1^]	S_mic_^f^ [m^2^g^−1^]	S_meso_^g^ [m^2^g^−1^]	V_t_^h^ [cm^3^g^−1^]	V_mic_^i^ [cm^3^g^−1^]	V_meso_^j^ [cm^3^g^−1^]
C-10	1.74	18.4	53.4	5.18	23.7	4.5	1480	1110	164	0.82	0.44	0.27
C-20	1.25	10.8	34.0	3.66	14.8	6.8	1420	1060	163	0.79	0.42	0.27
C-30	1.21	9.16	25.3	3.08	11.7	7.9	1140	845	136	0.64	0.34	0.25
C-45	1.11	7.41	25.7	3.02	11.2	8.2	1310	970	160	0.75	0.39	0.30
C-60	0.865	4.80	22.4	2.26	9.23	12.7	1240	910	156	0.71	0.36	0.29
C-90	0.813	6.11	21.0	2.20	9.75	12.8	480	290	99	0.35	0.11	0.20

^a^D_v_10 represents the particle size at which 10% of the particles are smaller on a volume basis. D_v_50 and D_v_90 represent the same factor with a threshold of 50% and 90% respectively.

^b^D[2,3] represents the surface-volume mean (also known as the Sauter mean).

^c^D[3,4] represents the mass-moment mean^27^.

^d^V < 1 μm represents the volume fraction of particles which are below the value of 1 μm.

^e^specific surface area calculated using the BET method.

^f^micropore surface area determined using the t-plot method.

^g^mesopore surface area determined from the adsorption branch of the isotherm using the BJH method.

^h^total pore volume calculated at P/P_0_ > 0.95.

^i^micropore volume determined using the t-plot method.

^j^mesopore volume determined using the BJH method.

**Table 2:** Characteristic capacitances and resistances.

Sample	c_CV_^a^ [Fg^−1^]	c_0.5_^b^ [Fg^−1^]	c_5.0_^c^ [Fg^−1^]	c_EIS_^d^ [Fg^−1^]	R_s_^e^ [Ω]	R_i_^f^ [Ω]
C-20	18.3	22.4	17.9	11.8	6.5	28.9
C-30	16.5	20.6	16.3	9.4	7.5	25.4
C-45	15.9	20.3	14.6	9.1	6.2	57.0
C-60	15.8	20.5	14.5	8.8	6.4	51.6

^a^specific capacitance determined from cyclic voltammetry at 25 mVs^−1^.

^b^specific capacitance determined from galvanostatic discharge at 0.5 Ag^−1^.

^c^specific capacitance determined from galvanostatic discharge at 5 Ag^−1^.

^d^specific capacitance determined from EIS at 10 mHz.

^e^series resistance determined from EIS where spectra cross real axis.

^f^ionic resistance from EIS.
