# Supplementary material for: Influence of Particle Size Distribution on the Performance of Ionic Liquid-based Electrochemical Double Layer Capacitors
Source: Sci Rep. 2016 Feb 25;6:22062. doi: 10.1038/srep22062 (PMC4766507; doi:10.1038/srep22062)
Supplement: Supplementary Information [file srep22062-s1.pdf]

Influence of Particle Size Distribution on the Performance of Ionic Liquid-based Electrochemical Double Layer Capacitors

Anthony J. R. Rennie,[*,a] *Vitor L. Martins,*[a,b] *Rachel M. Smith*[a] *and Peter J. Hall*[a]

[a] Chemical and Biological Engineering, University of Sheffield, Sir Robert Hadfield Building, Mappin Street, Sheffield S1 3JD, England, UK

[b] Instituto de Química, Universidade de São Paulo - C.P. 26077, CEP 05513-970, São Paulo, SP, Brazil

Supplementary Information

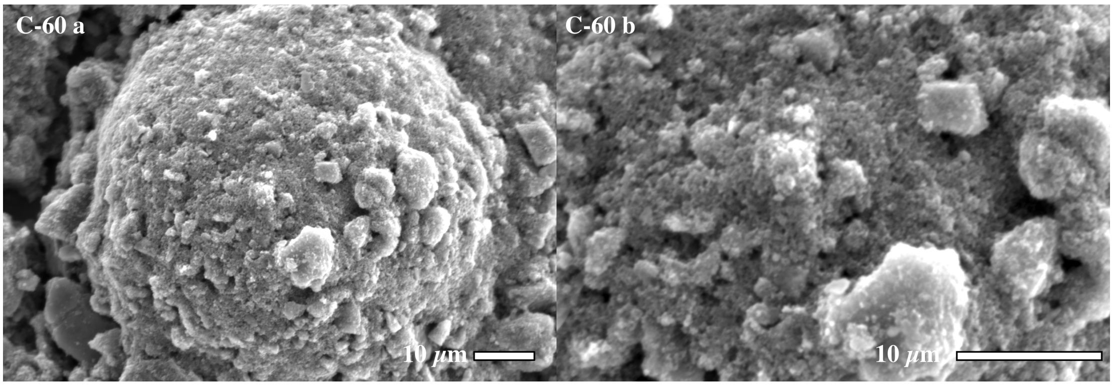

Figure 1S: (a) SEM images of the C-60 electrode at x1000magnification and (b) x2500 magnification

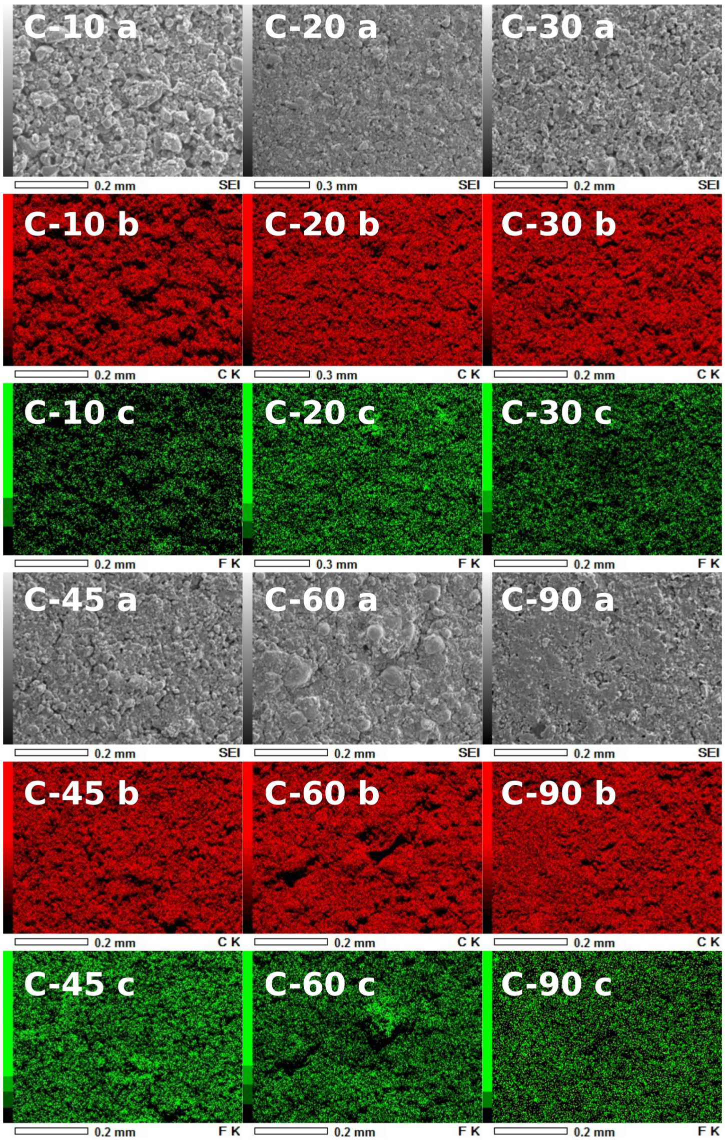

Figure 2S: SEM images (a) and EDX mapping of Carbon (b) and Fluorine (c) of electrodes coatings at 200 times of magnification.

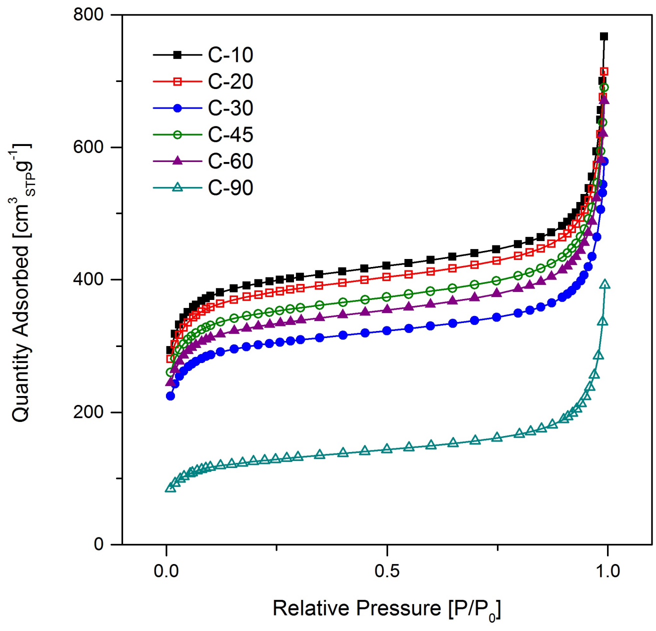

Figure 3S: Nitrogen adsorption isotherms of milled carbon materials at -196ºC. (Desorption isotherms not shown for reasons of clarity; no significant hysteresis was observed)
